# Editorial: The Role of Innate Lymphoid Cells in Mucosal Immunity

**DOI:** 10.3389/fimmu.2020.01233

**Published:** 2020-06-17

**Authors:** Jessica G. Borger, Graham Le Gros, Joanna R. Kirman

**Affiliations:** ^1^Department of Immunology and Pathology, Central Clinical School, Monash University, Alfred Research Alliance, Melbourne, VIC, Australia; ^2^Allergic & Parasitic Diseases Programme, Malaghan Institute of Medical Research, Wellington, New Zealand; ^3^Department of Microbiology & Immunology, University of Otago, Dunedin, New Zealand

**Keywords:** ILC, NK cell, mucosal, immunity, lung, gastrointestinal tract, genitourinary tract

Innate Lymphoid Cells (ILCs) are tissue-resident innate cells that are deeply integrated within mucosal tissues and play a critical role in tissue homeostasis and inflammatory processes. The collection of articles in this Frontiers Research Topic investigates and discusses the new and emerging roles of ILCs in mucosal immunity.

The field of ILC biology has increased exponentially in recent years. Although the scientific community had been aware of the existence of certain innate cells of lymphoid origin for many years, it has only been in the past decade that the full extent of the ILC family and their wide-reaching biological roles has been recognized. As the field expanded, to avoid confusion due to the different names being used in the literature for the same ILC subsets, in 2013 it was proposed that ILCs should be divided into three broad lineages based on specific transcription factor expression and the cytokines they produce. Group 1 (ILC-1) express *T-bet* and produce IFN-γ; Group 2 (ILC-2) express *GATA-3* and produce IL-5, IL-4, and IL-13; and Group 3 (ILC-3) express *ROR*γ*t* and produce IL-17 and IL-22 (1). These groups parallel the transcriptional and functional characteristics of T helper 1 (Th1), Th2, and Th17 conventional T cells, respectively. Like T helper subset plasticity, there is heterogeneity within the different ILCs groups, and some overlap between the subsets.

When comparing studies, it is important to recognize that methods to identify and categorize ILCs and their subsets vary from laboratory to laboratory. The lineage cocktail used to identify non-ILCs is the first potential source of variation between studies. This may result in contamination of ILCs and account for differences in the numbers of ILCs in different studies (2). The molecules used to categorize ILCs also vary and can greatly influence the number of cells identified in each subset; some studies use transcription factors to categorize ILCs, whilst others rely on the presence or absence of specific cell surface molecules.

Unlike their adaptive counterparts, activation of ILCs is not modulated by genetically rearranged antigen receptors. Rather, in the absence of conventional antigen recognition, ILCs respond directly or indirectly to a diverse array of pathogen- and host-derived stimuli including stress-related neuropeptides and hormones, cytokines, and metabolites (Jacquelot et al.). Due to their integration in tissues at barrier surfaces, and their ability to respond directly to signals without the requirement for antigen presentation, ILCs are poised to respond rapidly to pathogens and tissue injury. This response is facilitated by a transcriptional program that enables phenotypic and functional plasticity of ILCs such that they can mediate and regulate tissue homeostasis, morphogenesis, metabolism, repair and regeneration.

ILCs are located throughout the body, though they are concentrated at barrier sites, including the skin and mucosal surfaces. ILCs in the skin are important for regulating and maintaining epithelial and stromal cells and maintaining skin barrier homeostasis. Although skin ILCs are not covered in this Research Topic, understanding how their unique effector functions may underlie their contribution to the pathogenesis of inflammatory skin disorders such as dermatitis, psoriasis and immunity remains an exciting area for future investigation.

This Research Topic comprehensively addresses the role of ILCs in maintaining tissue homeostasis at mucosal sites including: the maternal and fetal compartments (Miller et al.; Vacca et al.), lung (Ardain et al.; Borger et al.), gut (Vojkovics et al.), adipose tissue (Bénézech and Jackson-Jones), and kidney (Cameron et al.). ILCs function within these mucosal tissues is regulated through a delicate and complex cross-talk between the immune system and the signals received from the physiological systems of the body. This cross-talk requires an exquisite balance to maintain homeostasis and when appropriate to respond to environmental cues to control infection and restore tissue damage (Jacquelot et al.).

During infection, ILCs are sensitive to diverse environmental signals and are critical for regulating responses to commensal bacteria (Castleman et al.) and pathogens including bacteria (Jacquelot et al.), helminthic parasites (Bouchery et al.; Löser et al.), and viruses (Ardain et al.; Panda and Colonna; Trittel et al.). Indeed, understanding how the dysregulation of ILC responses can lead to chronic inflammatory diseases including: allergy (Helfrich et al.; Panda and Colonna) and asthma (Ardain et al.; Borger et al.), chronic obstructive pulmonary disease (COPD) (Ardain et al.; Borger et al.; Panda and Colonna), and pulmonary fibrosis (Ardain et al.); as well as autoimmune diseases such as inflammatory bowel disease (IBD) (Vojkovics et al.), colitis (Panda and Colonna) and Crohn's disease, is a consistent theme within this Research Topic. ILCs also influence the development of mucosal tumors, such as colorectal cancer (Loyon et al.). Some ILC subsets play a pro-tumorigenic role; others appear to have a protective role through promotion of anti-tumor responses or by limiting damage to tissue (Loyon et al.). Although many mechanisms and fine details of the interplay between ILCs and their environment remain to be elucidated, it is clear that ILCs are critical for protecting mucosal barriers against communicable and non-communicable disease.

## Regulation of ILCs

The physiological regulation of ILCs in the maintenance of tissue homeostasis in mucosal sites is reliant on the exquisitely sensitive sensing of host-derived peptide and non-peptide signals that enable ILCs to integrate the physiological systems of the body including nervous, endocrine, digestive and reproductive systems. Jacquelot et al.. reviews recent advances in our understanding of the mechanisms by which ILCs are regulated by physiological signals. Jacquelot et al. evaluates how ILCs are able to integrate these signals to maintain homeostasis and prevent immunopathology without compromising infection control.

One such host-derived signal is S1P, a blood-borne bioactive lysosphingolipid which binds to the S1P receptor 1 (S1PR1) to promote lymphocyte egress from the secondary lymphoid organs. Until recently, the effects of S1P and pharmacological agonists of S1PR1 on ILCs was unknown. Such agonists include fingolimod, an immunomodulatory drug approved to modify the autoimmune disease multiple sclerosis (MS). In this Research Topic, Eken et al. reveals that ILCs express S1P1R and administration of S1P agonists including fingolimod, drives ILC-penia in MS patients and decreases ILC accumulation in the secondary lymphoid organs of mice. Notably, long-term administration of fingolimod in mice led to decreased ILC3s in the small intestine. This study characterizes a further mechanism by which ILCs can sense and respond to host-derived signals to maintain tissue homeostasis.

## ILCs in the Respiratory Tract

The lung is considered a non-lymphoid mucosal organ comprising an innate cell network of epithelial cells, macrophages, and dendritic cells, all crucial for maintenance of immune homeostasis and the initiation of inflammatory processes. In addition, ILCs, which although only comprise a small proportion of the total immune cells in the lung, have been shown to promote lung homeostasis and contribute to disease processes. In mice, ILC2s are the predominant ILC subset in the lung (3) and in this Research Topic, Helfrich et al., provides a comprehensive review on the contribution of ILC2s to allergic airway inflammation including allergic asthma, obesity-induced asthma, and rhinosinusitis.

Helfrich et al. considers the effect of experimental and therapeutic strategies that are employed to ameliorate respiratory inflammation on ILC responses in the lung. Corticosteroids and β2-agonists are common treatments for asthmatic patients and β2 adrenergic receptors are expressed by both human and murine ILC2s. The ILC2s respond β2-agonists by reducing proliferation and effector function, suggesting that targeting ILC2s early in disease could also block initiation of respiratory inflammation and redirect ILC2 activity Helfrich et al..

Although the role of ILC2s in Th2 allergic airway responses is well-supported, the contributions of ILCs to chronic lung inflammation are poorly understood. In this issue Borger et al. and Panda and Colonna review the contribution of ILCs to chronic pulmonary diseases. Dysregulation of ILCs and their ability to respond to changing local tissue environmental cues by altering their traits and functional attributes, contributes to the immunopathology underlying COPD, asthma, and chronic rhinosinusitis (Borger et al.; Panda and Colonna). The dysregulation of ILCs becomes even more pronounced during disease exacerbations, which are common in COPD and asthma. The major cause of disease exacerbation is viral infection. During viral infection, ILC2-activating cytokines, IL-25 and IL-33, are released by airway epithelial cells upon viral infection, enhancing type 2 responses and the recruitment of ILC2s.

ILC1s also contribute to the clearance of murine influenza infections of the lung. Trittel et al. provide evidence that ILC1s can be modulated via activated iNKT cells, to improve functional responsiveness. Notably, heightened cytokine production appeared restricted to the ILC1s that expressing checkpoint molecules on their surface. This suggests the exciting possibility that control of influenza infection and potentially other rhinovirus infections could be modulated by immunotherapeutic interventions.

In humans, by using cell surface markers to categorize ILCs, ILC3s, rather than ILC2s, are the most prevalent subset in lung tissue (4). ILC3 secretion of IL-17A and IL-22 has prompted investigations into their involvement in inflammatory and infectious diseases. Although the importance of ILC3s as a source of GM-CSF in the lung remains unknown, its role in allergic airway disease, antimicrobial pulmonary host defense function, and surfactant homeostasis is recognized. Together, this lends ILC3s in humans to have a pronounced capacity to drive inflammation and repair in numerous infectious and autoimmune diseases including: tuberculosis, bacterial pneumonia, asthma, COPD, and pulmonary fibrosis. In their review, Ardain et al. highlight the intriguing possibility of ILC3s as a therapeutic target for human pulmonary disorders.

In addition to the critical role tissue-resident ILCs provide in lung homeostasis and during inflammation, the contribution of other innate-like unconventional αβ- and γδ-T cells including MR1-restricted MAIT cells and CD1d-restriced NKT cells is now emerging in pulmonary immunity. Borger et al. evaluate the role of these tissue-resident unconventional T cells in chronic pulmonary disorders and interrogate their interplay and functional overlap with ILCs, asking whether these cell subsets co-regulate one another or function independently. The heterogenous nature and functional plasticity of ILCs and their deep integration within the lung tissue, would suggest that unconventional T cells can influence ILC responses during inflammation and this important relationship remains to be addressed.

## ILCs in the Gastrointestinal Tract

ILCs are involved in normal mucosal lymphoid tissue formation as well as in inflammation and epithelial regeneration in IBD. Nkx2-3 is a transcriptional regulator of MAdCAM-1, which is a susceptibility factor for IBD in humans, and is associated with neogenesis of lymphoid tissue in inflamed intestines. In this Research Topic, Vojkovics et al., explore the role of Nkx2-3 in the organogenesis of the solitary intestinal lymphoid tissues involving type 3 innate lymphoid cells (ILC3). It was found that the total absence of MAdCAM-1 partially impaired postnatal seeding of ILC3s in the intestine and caused partial blockade of solitary intestinal lymphoid tissues maturation, suggesting other adhesion molecules may compensate for the intestinal homing of ILC3s in the absence of MAdCAM-15(Vojkovics et al.).

ILCs are also important regulators of adipose tissue function, and in particular ILC2s in the adipose tissue of mucosal sites, such as the mesenteries, and play a critical role in innate B cell activation and antibody production. Bénézech and Jackson-Jones discuss the role of adipose tissue ILCs in the lean state and in obesity, including their link to metabolic dysfunction. Fat associated lymphoid clusters are found throughout the pleural and peritoneal cavities and the ILCs located within these are critical for initiation of immune responses near mucosal sites and for maintaining intestinal barrier function.

Another important means by which ILCs promote gut homeostasis is via IL-22 produced by ILC3s, which enhances epithelial barrier integrity. When the epithelial barrier is compromised, either due to inflammation or infection, microbes can translocate from the gut lumen into the lamina propria, and in mouse models this induces pro-inflammatory cytokine production from ILC3. Castleman et al. used an *in vitro* human lamina propria mononuclear cell model to investigate whether enteric commensal and pathogenic bacteria drives human ILC3 to produce pro-inflammatory cytokines such as IFN-γ. Although IFN-γ was not induced in this *in vitro* model by commensal or pathogenic bacteria, the model provided insight into the mechanisms by which commensal bacterial drive IL-22 production by ILC3. Enteric commensal bacteria cannot directly induce cytokine production by ILC3s, but rather acted on mononuclear cells to produce cytokines that in turn enhanced IL-22 production by ILC3s (Castleman et al.).

In addition to their role in lymphoid and adipose tissue formation and homeostasis in the gastrointestinal tract, ILCs are important for controlling infection. ILC2s were first discovered in experimental studies of intestinal helminth infection (Bouchery et al.). ILC2s sense a wide array of stimuli from helminths, allergens and some bacteria as well as host-derived danger signals induced by tissue damage and release of chemical messengers and other molecules from stressed cells. As helminths mature within the gut tissue, they drive epithelial and mucosal defenses from the host to activate tissue resident intestinal ILC2s, that closely associate with the sensory enteric neuron network. In this Research Topic Löser et al. and Bouchery et al. discuss how ILC2s are embedded in a network of barrier responses during helminth infection, including luminal and epithelial crosstalk, the translation of alarmin signals from host epithelium and eicosanoid mediators, as well as the essential role of ILCs in neuro-immune responses in the lung and gut respectively.

ILCs can play a dual role in the context of tumors, displaying pro- or antitumour effects dependent on the ILC subset and type of cancer. Loyon et al. reveal that treatment-free naïve metastatic colorectal cancer patients have increased peripheral ILCs skewed toward the ILC1 subset and in particular CD56^+^ ILC1-like cells, which negatively correlate with the anti-tumor CD4 T cell response. Of interest, this study also highlights that although different chemotherapy routines did not alter ILC numbers, there was potential to modulate ILC1 subsets such that the balance of ILC1 and CD56^+^ ILC1-like cells could be reversed, with ILC2 refractory to the effects of treatment, highlighting the importance of considering the ILC compartment in treatment and monitoring of cancer (Loyon et al.).

## ILCs in the Genitourinary Tract

In this Research Topic Cameron et al. show that in mice, a higher proportion of CD45^+^ cells are ILC2s in the kidney than in the lungs. The kidney resident ILC2s constitutively produced IL-5 under homeostatic conditions. Others have found that artificially-induced ILC2s can reduce the severity of experimental renal injury. In this study, the impact of ILC2-deficiency on ischemia-reperfusion injury was investigated, and surprisingly the extent of renal injury was similar whether or not ILC2s were present (Cameron et al.). Thus, although ILC2s can ameliorate renal injury, other compensatory mechanisms exist in their absence.

During pregnancy there is an careful balance between maternal and fetal immune systems, with reviews by Miller et al. and Vacca et al. discussing the potential role of ILCs within the maternal and fetal compartments Miller et al. provide a broad characterization of ILC populations within the uterus, decidua, fetal tissues, and amniotic cavity and highlights the many differences between human and mouse ILC subsets in these locations. For example, although ILC1, ILC2, and ILC3 are present in the mouse uterus prior and during pregnancy, they have only been detected in the non-pregnant endometrium in humans demonstrating a vast disparity in the requirement of ILCs for shaping a successful pregnancy between humans and mice. Moreover, in the decidua which contains decidual NK cells, tissue-resident NK cells and ILC3s, Vacca et al. consider how the changing decidual microenvironment has influenced the plasticity and function of ILCs during early pregnancy, and reflects that their involvement in the establishment and maintenance of pregnancy remains unanswered.

## Conclusion

The collection of original research articles and reviews in this Research Topic demonstrated the ability of ILCs to modulate the course of infection, inflammatory processes, and cancer across different mucosal barriers in both mice and humans. The important role ILCs have in supporting homeostasis at these barrier sites was also highlighted. Although there have been enormous advances in our understanding of ILCs over the past decade, much remains to be discovered, including: the signals that activate different ILC subsets at each site; the redundancy and plasticity of ILCs and ILC subsets at different anatomical locations; and how ILCs can be modulated in the clinic to improve outcomes.

## Author Contributions

JB and JK jointly recruited authors and scope for this issue. JB, GL, and JK reviewed submissions and contributed to the writing of the forward to this issue. All authors contributed to the article and approved the submitted version.

## Conflict of Interest

The authors declare that the research was conducted in the absence of any commercial or financial relationships that could be construed as a potential conflict of interest.
